# Anticoagulant activity of singlet oxygen released from a water soluble endoperoxide by thermal cycloreversion†

**DOI:** 10.1039/d1ra02569d

**Published:** 2021-04-19

**Authors:** Meina Liu, Esma Ucar, Ziang Liu, Lei Wang, Li Yang, Jiawei Xu, Engin U. Akkaya

**Affiliations:** State Key Laboratory of Fine Chemicals, Dalian University of Technology 2 Linggong Road 116024 Dalian China leiwang@dlut.edu.cn eua@dlut.edu.cn; Department of Pharmaceutical Science, School of Chemical Engineering, Dalian University of Technology 2 Linggong Road 116024 Dalian China; Department of Chemistry, Bilkent University 06800 Ankara Turkey; College of Pharmacy, Liaoning University of Traditional Medicine 110847 Shenyang China

## Abstract

Singlet oxygen generated by photosensitization has limited potential *in vivo* due to light attenuation in tissues. However, controlled chemical generation of this reactive oxygen species is likely to open new therapeutic spaces to explore. The fact that its activity is limited by the rate of cycloreversion reaction and the diffusion distance of the excited state molecular oxygen species, is a clear advantage, considering the serious side effects of off-target anticoagulants. In this work, we present novel 1,4-naphthalene endoperoxides as potential anti-coagulant agents due to thermal release of singlet oxygen.

## Abstract 2

Singlet oxygen generated by the cycloreversion of endoperoxides has anti-coagulant activity.

## Graphical abstract

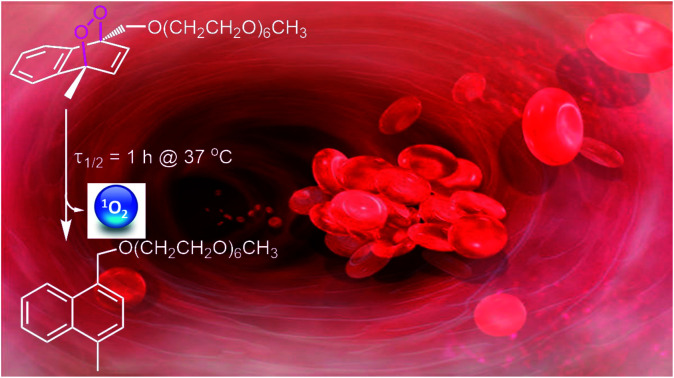

## Introduction

Among reactive oxygen species, singlet oxygen has a unique place as it is the most reactive, and short-lived.^1^ Other than being a useful chemical agent,^2^ it is known to be primarily responsible for the photodynamic action^3^ and various critical biological processes, including regular maintenance.^4^

Within the last decade, there has been accumulation of evidence^5^ suggesting a role for singlet oxygen in hemostasis, which is the system of generation and destruction of thrombi, which involve opposing actions of coagulation and thrombolysis. The cellular part of the hemostatic process involves the thrombocytes and endothelial cells for coagulation and the polymorphonuclear granulocytes (PMN) for thrombolysis. PMN are known^6^ to generate singlet oxygen enzymatically by the action of NADPH-oxidase and myeloperoxidase. The short lifetime of singlet oxygen, may help to put a spatio-temporal limit to anticoagulant action.

Singlet oxygen can be generated by the intermediacy of photosensitizers under irradiation of ground state molecular oxygen, or alternatively by chemical reactions of phosphine/phosphite ozonides;^7^ also, molybdate,^8^ chloramine and HOCl reactions with H_2_O_2_.^9^ The chemical methods mentioned above all require strongly oxidant inorganic species which would not allow much flexibility in their utilization. However, in recent years aromatic endoperoxides,^10^ with their tunable rates of singlet oxygen release,^11^ attracted attention as they appear to be more amenable for biological utilization.

Both photogenerated singlet oxygen^12^ and chemically generated (hypochlorite/chloramine reaction) singlet oxygen^13^ was shown to have thrombolytic action *in vitro*. Unfortunately, these two methods will have limited value *in vivo*, because of the strong light attenuation in tissues, and impractical nature of producing large concentrations of hypochlorite and chloramine or hydrogen peroxide, on demand. Thus, we posit that the most viable option is to generate singlet oxygen by the thermal cycloreversion of aromatic endoperoxides.

## Experimental

### Materials and instrumentation

All commercial chemicals were used as supplied unless otherwise indicated. Anhydrous solvents were obtained from a Solvent Purification System. Column chromatography was performed using silica gel (200–300 mesh). ^1^H and ^13^C NMR spectra were recorded on Bruker Avance II 400 MHz or Bruker Avance III 500 MHz. Signal splitting patterns were described as singlet (s), doublet (d), triplet (t), quartet (q) and multiplet (m) with coupling constants (*J*) in hertz (Hz). High resolution mass spectra (HRMS) were recorded with an Agilent mass spectrometer. Reactions were monitored by thin-layer chromatography using Merck TLC Silica gel 60 F254.

#### Synthesis of compound 2

1,4-Dimethylnaphthalene (1) (0.44 g, 2.8 mmol, 1 equiv.) was dissolved in dichloromethane (16.0 mL) and degassed with argon. Under an argon atmosphere, *N*-bromosuccinimide (2 equiv.) and benzoyl peroxide (0.07 equiv.) were added and the suspension was degassed to give a yellow suspension. The reaction mixture was heated under reflux argon atmosphere for 5 hours. The reaction mixture was cooled to room temperature and then washed with 2.0 M HCl (2 × 15 mL), 2.0 M NaOH (2 × 20 mL), brine, and dried with MgSO_4_. The solvent was evaporated to yield an off-white powder as the crude product, which was purified by column chromatography with 1 : 1 (vol/vol) dichloromethane/petroleum ether to yield the desired product in 50% yield. Used without further purification.

#### Synthesis of compound 3

Hexaethyleneglycol monomethyl ether (2.0 equiv.) was dissolved in dry THF (5.0 mL) under argon atmosphere. After that, sodium hydride (2.0 equiv.) was added and the mixture was stirred under reflux for 1 h. Next compound 2 (0.040 g, 0.17 mmol, 1.0 equiv.) dissolved in THF (1.0 mL) was added. The reaction mixture was stirred for 12 h under reflux. Then, the crude mixture was concentrated in vacuum. The mixture was diluted with CH_2_Cl_2_, washed with H_2_O and the solvent was removed in vacuum. The residue was purified by column chromatography with 10 : 1 (vol/vol) CH_2_Cl_2_/MeOH to get the desired product (36.0 mg, 0.048 mmol) in 50% yield. ^1^H NMR (400 MHz, CDCl_3_) *δ* 8.21–8.19 (m, 1H), 8.06–8.03 (m, 1H), 7.59–7.54 (m, 2H), 7.40 (d, *J* = 8.0 Hz, 1H), 7.30–7.28 (m, 1H), 5.01 (s, 2H), 3.69–3.65 (m, 24H), 3.40 (s, 3H), 2.71 (s, 3H); ^13^C NMR (100 MHz, CDCl_3_) *δ* 126.5, 125.9, 125.8, 125.6, 124.8, 124.6, 72.0, 71.9, 70.70, 70.65, 70.62, 70.61, 70.59, 70.58, 70.53, 69.3, 59.0, 19.5; HRMS (ESI) *m*/*z* calcd for C_25_H_38_O_7_Na [M + Na]^+^ 473.2510, found 473.2513.

#### Synthesis of compound 4

Compound 3 (0.02 g, 0.04 mmol) was dissolved in D_2_O (1.0 mL). The reaction mixture was cooled to 0 °C. Methylene blue was added into the solution and mixture was stirred for 8 hours under oxygen atmosphere. During the reaction, 630 nm lamp (red light irradiation) was used. After removal of the methylene blue by mixing with 0.5 g cation exchange resin (Chelex 100, Na^+^ form) and filtration. ^1^H NMR (400 MHz, D_2_O) *δ* 7.43–7.29 (m, 4H), 6.89 (d, *J* = 8.0 Hz, 1H), 6.80 (d, *J* = 8.0 Hz, 1H), 4.43–4.32 (m, 2H), 3.65–3.55 (m, 24H), 3.28 (s, 3H), 1.84 (s, 3H).

#### Synthesis of compound 5

1,4-Dimethylnaphthalene (1) (0.44 g, 2.8 mmol, 1 equiv.) was dissolved in dichloromethane (16 mL) and degassed with argon. Under an argon atmosphere, *N*-bromosuccinimide (3 equiv.) and benzoyl peroxide (0.1 equiv.) were added and the suspension was degassed to give a yellow suspension. The reaction mixture was heated under reflux argon atmosphere for 9 hours. The reaction mixture was cooled to room temperature and then washed with 2.0 M HCl (2 × 15 mL), 2 M NaOH (2 × 20 mL), brine, and dried with MgSO_4_. The solvent was evaporated to yield an off-white powder as the crude product, which was purified by column chromatography with 1 : 1 (vol/vol) dichloromethane/petroleum ether to yield the desired product in 80% yield. ^1^H-NMR (400 MHz, CDCl_3_) *δ* 8.22–8.21 (m, 2H), 7.68–7.66 (m, 2H), 7.49 (s, 2H), 4.94 (s, 4H).

#### Synthesis of compound 6

Hexaethylene glycol monomethyl ether (4 equiv.) was dissolved in dry THF (5.0 mL) under argon atmosphere. After that, sodium hydride (4.0 equiv.) was added and the mixture was stirred under reflux for 1 h. Next compound 5 (0.0526 g, 0.17 mmol, 1.0 equiv.) dissolved in THF (1.0 mL) was added. The reaction mixture was stirred for 12 h under reflux. Then, the crude mixture was concentrated in vacuum. The mixture was diluted with CH_2_Cl_2_, washed with H_2_O and the solvent was removed in vacuum. The residue was purified by column chromatography with 10 : 1 (vol/vol) CH_2_Cl_2_/MeOH to get the desired product (36 mg, 0.048 mmol) in 30% yield. ^1^H-NMR (500 MHz, CDCl_3_) *δ* 8.17–8.15 (m, 2H), 7.54–7.53 (m, 2H), 7.44 (s, 2H), 5.00 (s, 4H), 3.68–3.64 (m, 44H), 3.55–3.53 (m, 4H), 3.37 (s, 6H); ^13^C NMR (125 MHz, CDCl_3_) *δ* 134.2, 132.1, 126.0, 125.8, 124.6, 72.0, 71.8, 70.69, 70.66, 70.62, 70.59, 70.58, 70.5, 69.5, 59.0; HRMS (ESI) *m*/*z* calcd for C_38_H_64_O_14_Na [M + Na]^+^ 767.4188, found 767.4188.

#### Attempted synthesis of compound 7

Compound 6 (0.020 g, 0.027 mmol) was dissolved in 1.0 mL D_2_O. The reaction mixture was cooled to 0 °C. Methylene blue was added into the solution and mixture was stirred for 8 hours under oxygen atmosphere. During the reaction, 630 nm LED lamps (red light irradiation) was used. After removal of the methylene blue by cation exchange resin, the crude product by analysed was shown to be an intractable mixture, made more difficult to handle by the short halflives of the endoperoxides (with 1,4 and 5,8-bridges, presumably).

## Results and discussion

**Fig. 1:**
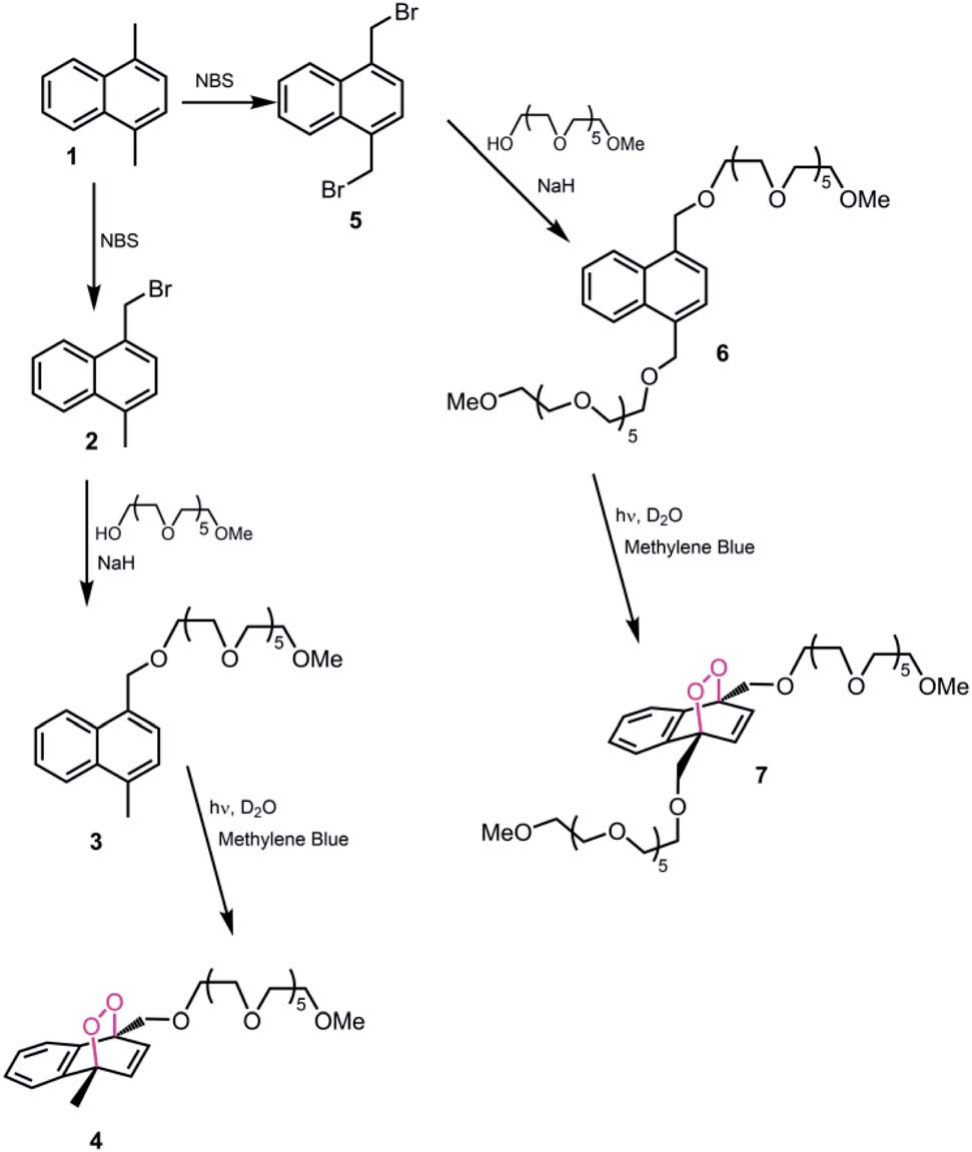
Synthesis of the PEG-functionalized, water soluble naphthalene endoperoxides.

Here in this work, we targeted the synthesis of PEG-functionalized naphthalene endoperoxides of the structures shown in Fig. 1.

**Fig. 2:**
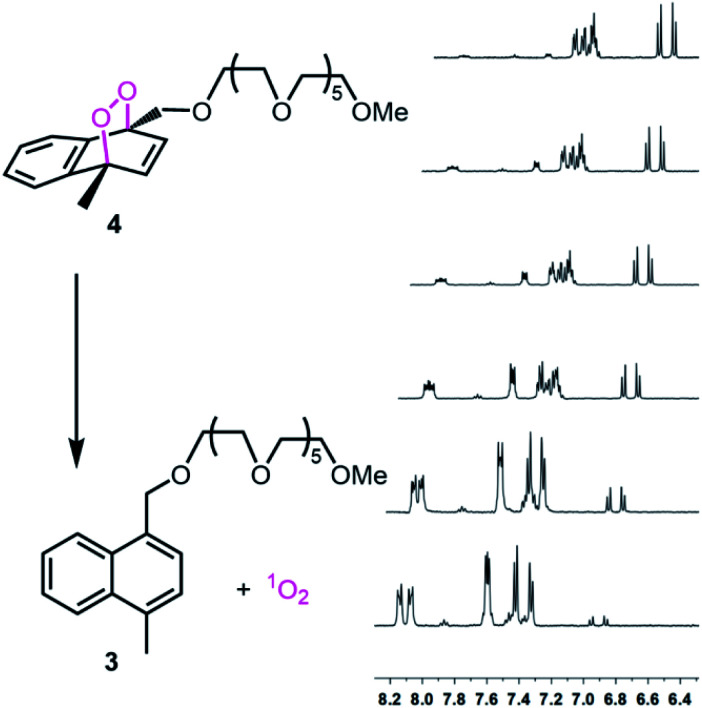
Gradual thermal conversion of the endoperoxide 4 (top spectrum) to its naphthalene precursor 3 at 37 °C in D_2_O. Spectra from top to bottom: 0, 10 minutes, 30 minutes, 1 h, 2 h, 3 h, respectively.

Bromomethyl and 1,4-(bis-bromomethyl)naphthalenes were reacted with hexaethyleneglycol monomethyl ether in the presence of NaH in THF. The products were purified by chromatography. The reaction with photogenerated singlet oxygen was carried out in D_2_O, using methylene blue as photosensitizer and a red LED array for light source at 4 °C. Endoperoxides were purified by column chromatography. Doubly-PEGylated naphthalene 7, did not react very well, and produce an intractable mixture of the desired endoperoxide 7 (15–20%), unreacted material 6 and 5,8-endoperoxide. Thus, we focussed on the mono-PEGylated compound 4 with high reaction yield (>98%). The half-life of the endoperoxide was determined by NMR in D_2_O, and found to be 53 minutes at 37 °C (Fig. 2). The NMR spectrum in the aliphatic region was crowded with PEG and methyl peaks, however aromatic region of the spectra can be used to study the rate of progression from the endoperoxide back to naphthalene core. The disappearance of the olefinic protons at 6.8–7.0 ppm was coupled to the emergence of the aromatic set at 8.0–8.2 ppm. This is in accordance with the expected changes on the ejection of singlet oxygen.

*In vitro* tests of anti-coagulant activity of the endoperoxide 4 at 37 °C were carried out using rat blood plasma. As a control, compound 3 was used to isolate the role of singlet oxygen precisely. Rat blood were collected into plastic tubes with sodium citrate (citrate/blood: 1/9, v/v) for plasma anticoagulation. After centrifugation at 3000 rpm for 10 minutes, 300 μL of plasma was mixed with 30 μL of endoperoxide with a final concentration of 0–10 mM and incubated for 30 min at 37 °C. Then, prothrombin time (PT), activated partial thromboplastin time (APTT), thrombin time (TT) and fibrinogen (FIB) were analyzed by a blood coagulation analyzer (Sysmex CA-500) with commercial kits following the manufacturer's instructions. The experiments were conducted in multiplicates (*n* = 3), and the results are presented as mean ± standard deviation (SD).

Activated Partial Thromboplastin Time (APTT) measures the overall speed at which blood clots by means of two distinct pathways, intrinsic pathway and the common pathway of coagulation. Partial thromboplastin time (PTT) measures a number of coagulation factors such as fibrinogen, prothrombin, proaccelerin, anti-hemophilic factor, Stuart–Prower factor, plasma thromboplastin antecedent, and Hageman factor. The prothrombin time (PT) is a measure of the clotting *via* the extrinsic pathway. TT and FIB measures specifically the rate of fibrin clot formation, which maybe more sensitive changes produced by singlet oxygen.

**Fig. 3:**
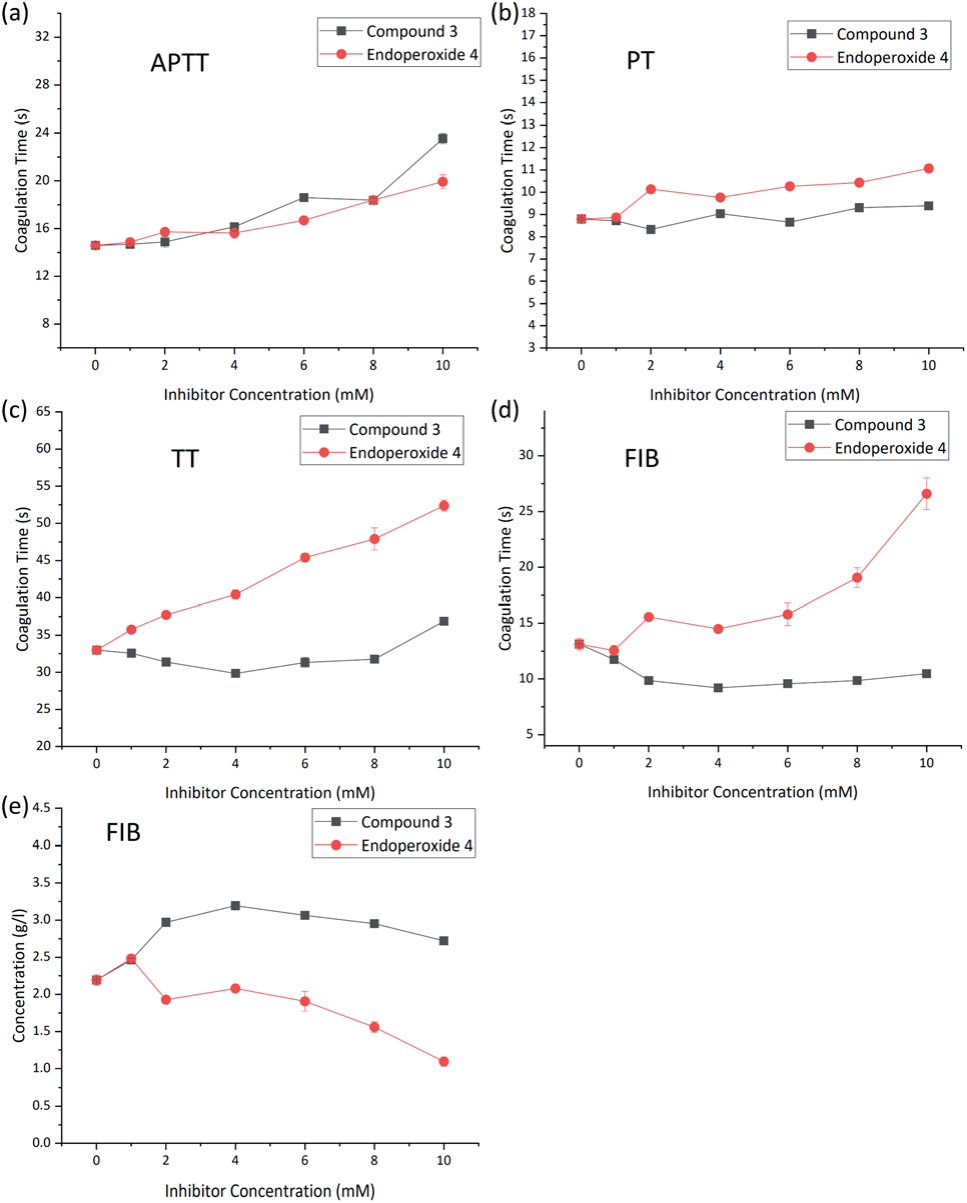
(a–e) Coagulation data and oxidative inactivation of fibrinogen. Treated plasma was incubated with 0–10 mmol L^−1^ (final concentration) compound 3 (control, black squares) or compound 4 (red diamonds) for 30 minutes (37 °C). Various coagulation parameters were acquired by blood coagulation analyzer.

The results were supportive of our expectations. Within the concentration range studied (0–10 mM) APTT (Fig. 3a) and PT (Fig. 3b) did not show significant changes, but TT (Fig. 3c) and FIB time (Fig. 3d), and the fibrinogen concentration changed in accordance with anti-coagulant activity (Fig. 3e).

While it is possible that the effect of singlet oxygen on hemostatic equilibrium can be, and most likely is multi-faceted, there is already literature data suggesting that fibrinogen is oxidized by singlet oxygen at the methionine sites, blocking the formation of polymeric fibrils and thus clotting. In addition, oxidized fibrinogen is converted by thrombin into a plasminogen activator. Plasminogen when hydrolytically activated is transformed into plasmin, which is the major factor for the breakdown to fibrin polymers.

## Conclusions

In summary, we present a water soluble endoperoxide with a potential to inhibit clotting and enhance thrombolysis. Our methodology is novel because of the fact that we use endoperoxides, which are more likely to be non-toxic at cell and organ level compared to hypochlorites. Optimized derivatives of these compounds are likely to find utility in a number of diseases that feature blood clots, such as pulmonary embolism, myocardial infarction, and stroke. The most common use would be for ischemic stroke, by either systemic administration or through the application of an arterial catheter directly to the site of the peripheral arterial thrombi. Our work in further assessment of the therapeutic potential of endoperoxide delivered singlet oxygen is in progress.

## Conflicts of interest

There are no conflicts to declare.

## Supplementary Material

RA-011-D1RA02569D-s001
