# Supplementary material for: Anticoagulant activity of singlet oxygen released from a water soluble endoperoxide by thermal cycloreversion
Source: RSC Adv. 2021 Apr 19;11(24):14513–6. doi: 10.1039/d1ra02569d (PMC8697772; doi:10.1039/d1ra02569d)
Supplement: RA-011-D1RA02569D-s001 [file RA-011-D1RA02569D-s001.pdf]

Electronic Supplementary Material (ESI) for RSC Advances.
This journal is © The Royal Society of Chemistry 2021

ELECTRONIC SUPPLEMENTARY INFORMATION

Anticoagulant activity of singlet oxygen released from a water soluble endoperoxide by thermal cycloreversion

Meina Liu,[ab] Esma Ucar,[c] Ziang Liu,[ab] Lei Wang,*[ab] Li Yang,[b] Jiawei Xu[d] and Engin U. Akkaya*[ab]

[a] State Key Laboratory of Fine Chemicals, Dalian University of Technology, 2 Linggong Road, 116024, Dalian, China.
[b] Department of Pharmaceutical Science, School of Chemical Engineering, Dalian University of Technology, 2 Linggong Road, 116024, Dalian, China.
[c] Department of Chemistry, Bilkent University, 06800 Ankara, Turkey
[d] College of Pharmacy, Liaoning University of Traditional Medicine, 110847 Shenyang, China.

^{1}H and ^{13}C NMR Spectra

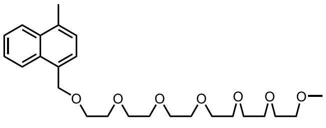

3

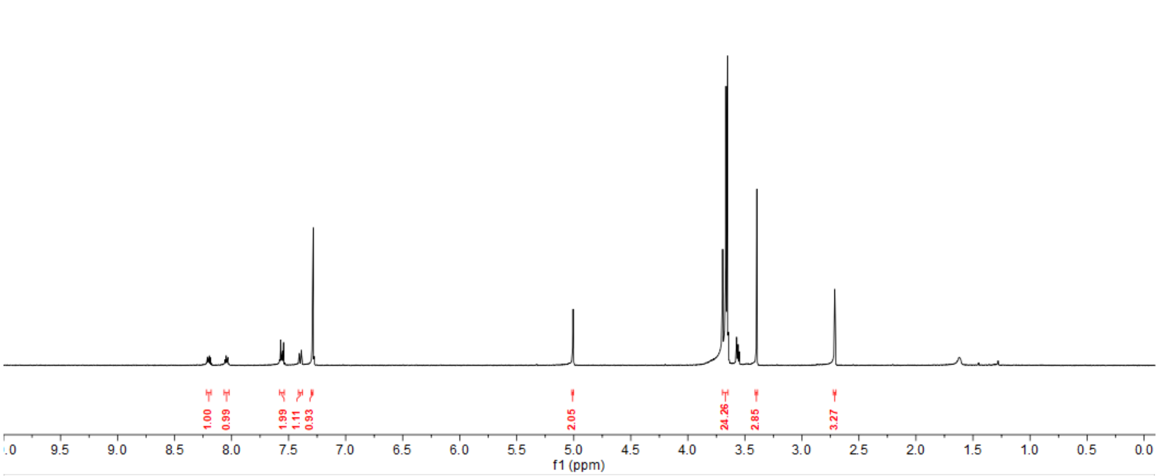
8.21
8.20
8.20
8.19
8.06
8.05
8.04
8.04
8.03
7.57
7.56
7.55
7.54
7.41
7.39
7.30
7.28
5.01
3.69
3.67
3.65
3.40
2.71
1.00
0.99
1.99
1.11
0.93
2.05
24.26
2.85
3.27
f1 (ppm)

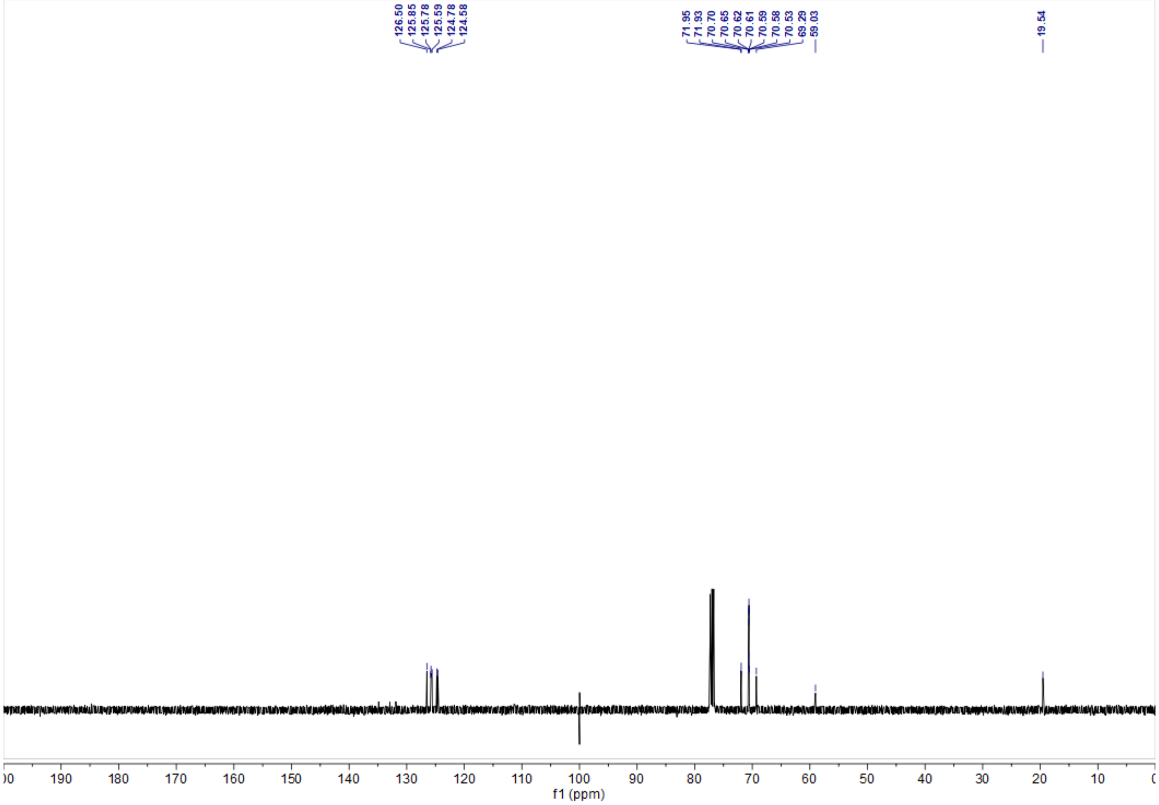
126.50
125.85
125.78
125.59
124.78
124.58
71.95
71.93
70.70
70.65
70.62
70.61
70.59
70.58
70.53
69.29
59.03
19.54
f1 (ppm)

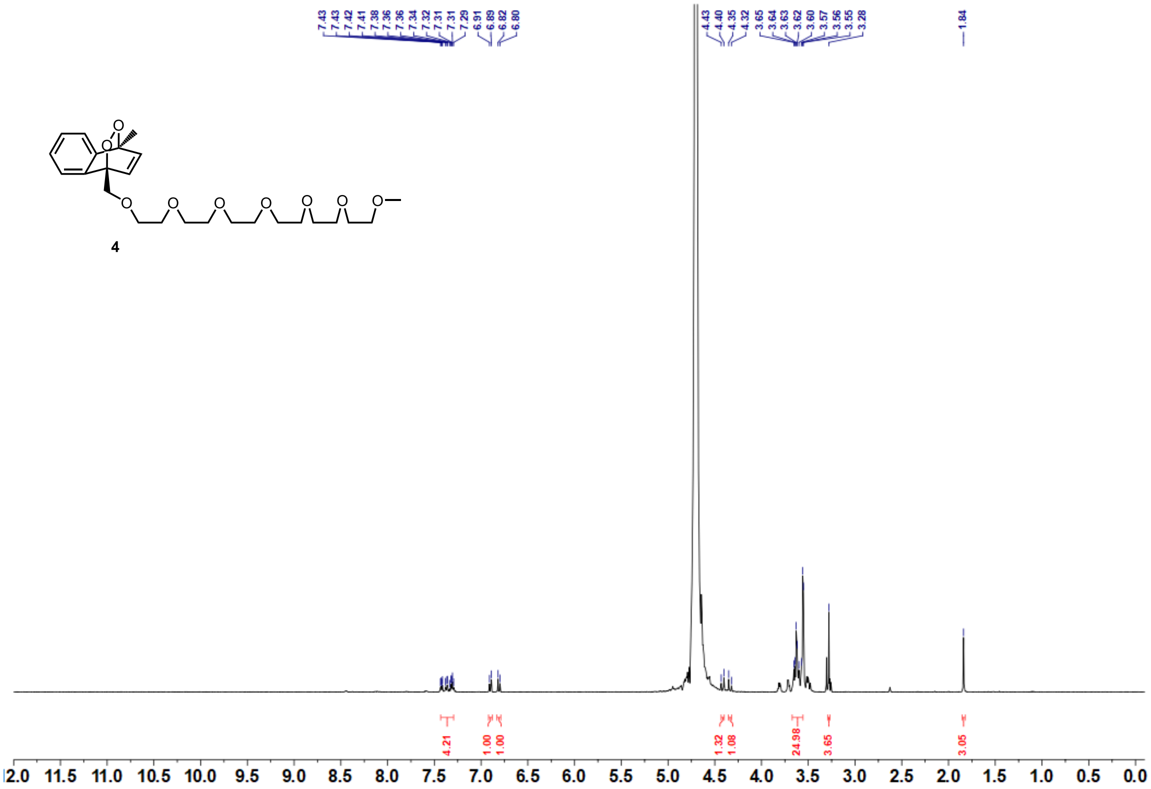
7.43 7.43 7.42 7.41 7.38 7.36 7.36 7.34 7.32 7.31 7.31 7.29 6.91 6.89 6.82 6.80
4.43 4.40 4.35 4.32 3.65 3.64 3.63 3.62 3.60 3.57 3.56 3.55 3.28
1.84
4.21 1.00 1.00 1.32 1.08 24.98 3.65 3.05
2.0 11.5 11.0 10.5 10.0 9.5 9.0 8.5 8.0 7.5 7.0 6.5 6.0 5.5 5.0 4.5 4.0 3.5 3.0 2.5 2.0 1.5 1.0 0.5 0.0

4

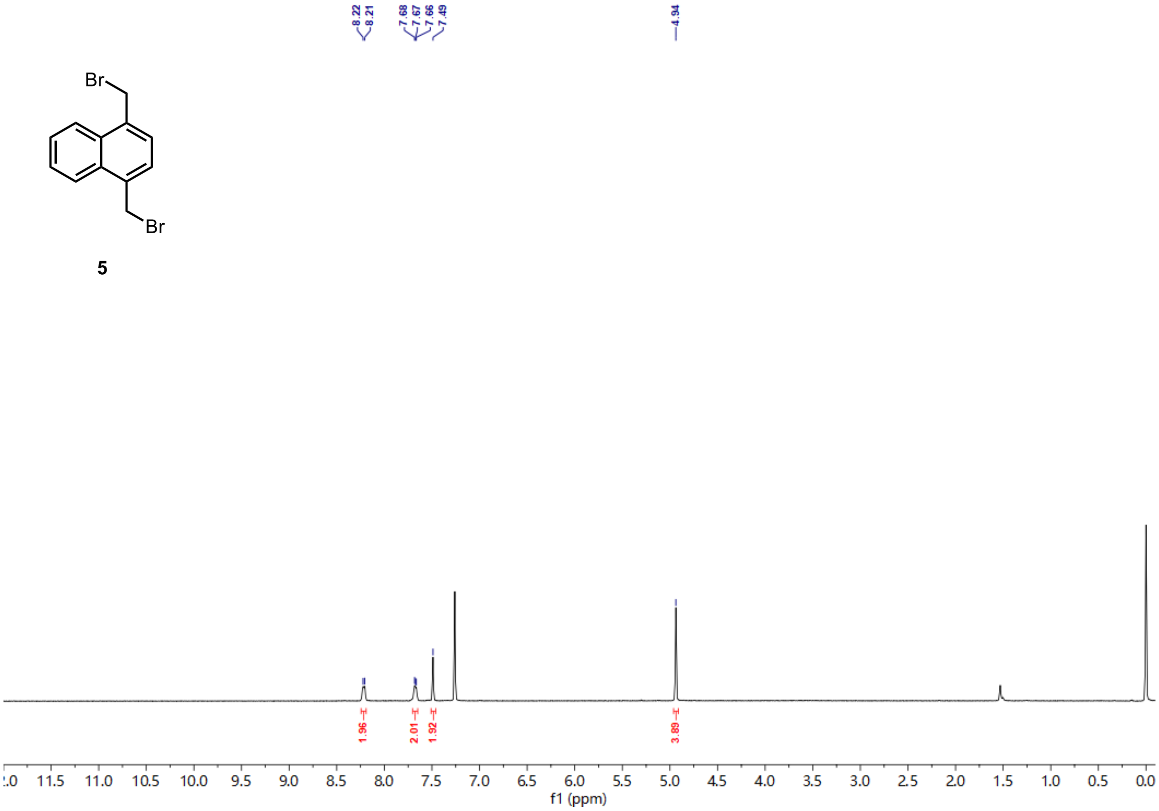
8.22 8.21 7.68 7.67 7.66 7.49
4.94
Br
Br
1.96 2.01 1.92 3.89
.0 11.5 11.0 10.5 10.0 9.5 9.0 8.5 8.0 7.5 7.0 6.5 6.0 5.5 5.0 4.5 4.0 3.5 3.0 2.5 2.0 1.5 1.0 0.5 0.0
f1 (ppm)

5

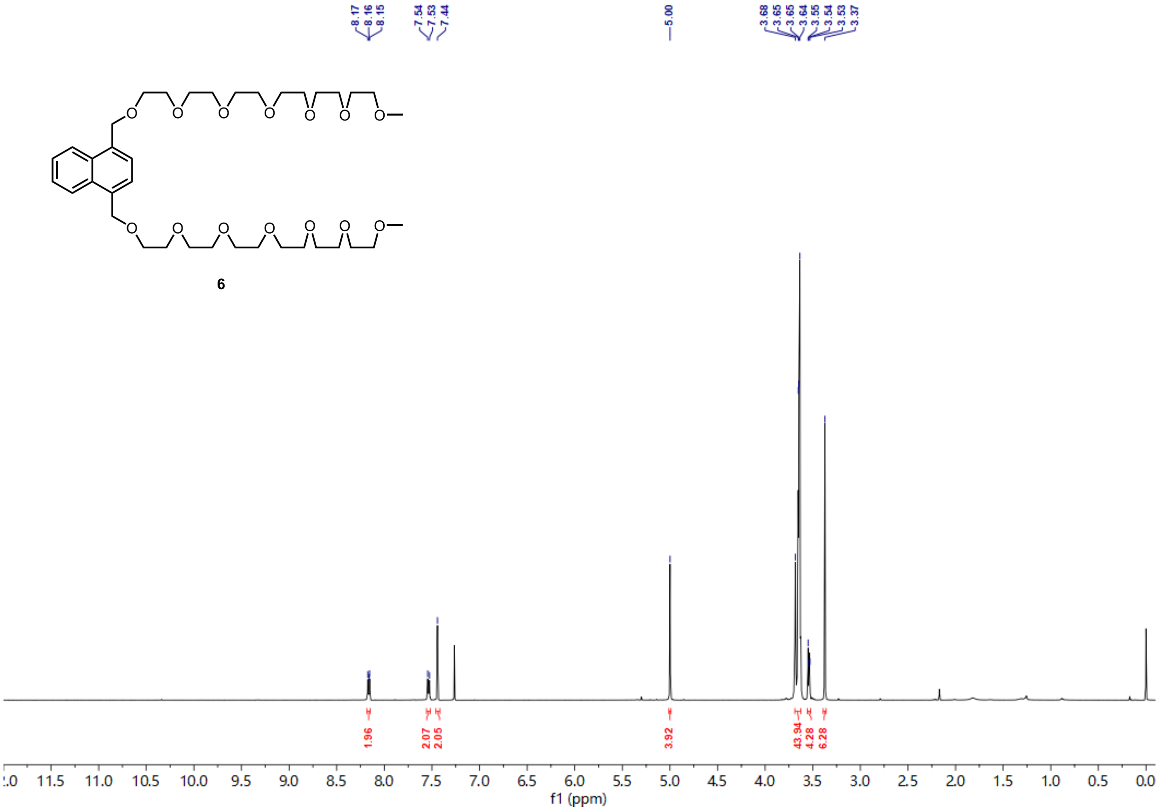

6
8.17
8.16
8.15
7.54
7.53
7.44
5.00
3.68
3.65
3.65
3.64
3.55
3.54
3.53
3.37
1.96
2.07
2.05
3.92
43.94
4.28
6.28
f1 (ppm)

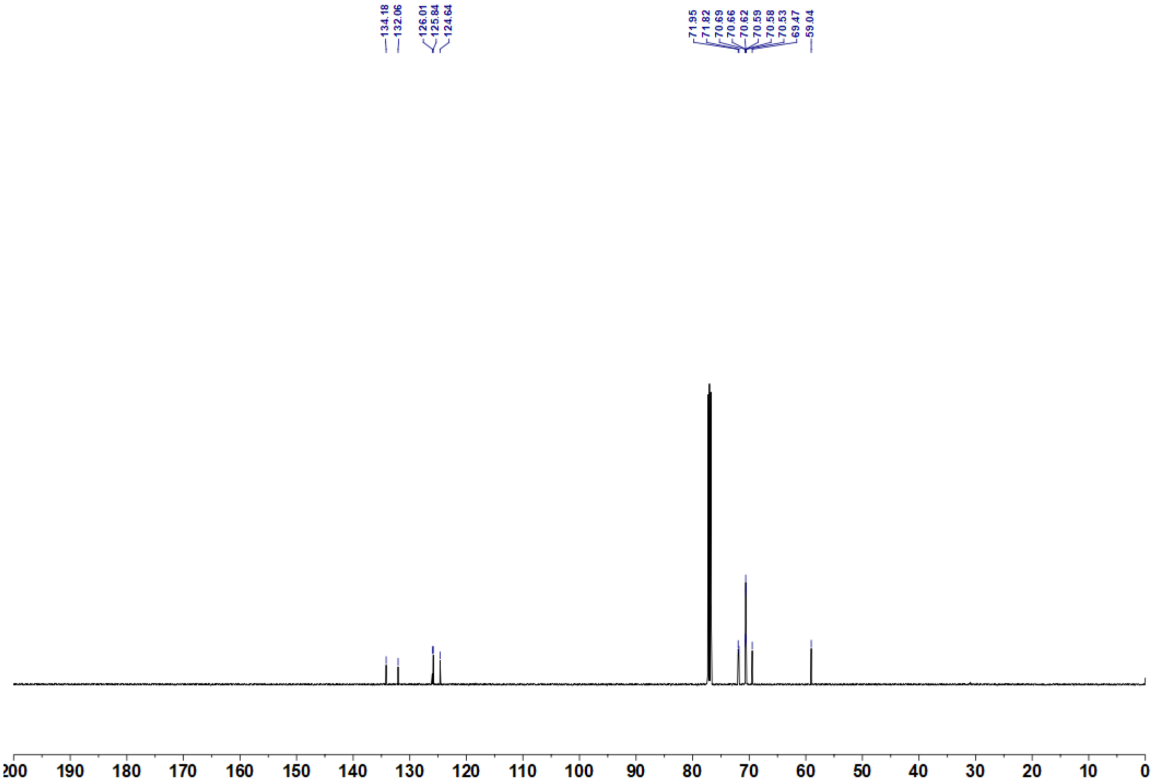

134.18
132.06
126.01
125.84
124.64
71.95
71.82
70.69
70.66
70.62
70.59
70.58
70.53
69.47
59.04
